# Response by Chirumbolo to “Rare Localized Adverse Reaction to Botulinum Toxin Type A in a Patient Without Allergic History: A Case Report” by Muner et al

**DOI:** 10.1093/asjof/ojag087

**Published:** 2026-05-12

**Authors:** Salvatore Chirumbolo

Muner et al reported a rare localized vesiculobullous inflammatory reaction after prabotulinumtoxinA and its subsequent improvement with oxygen–ozone therapy (OOT), thereby drawing attention to both the safety profile of botulinum toxin formulations and the possible therapeutic value of ozone in refractory inflammatory injury.^1^ Although botulinum toxin is widely used and generally considered safe, this case is a reminder that adverse reactions may arise not only from immune mechanisms but also from procedural variables, including injection depth, reconstitution, aseptic technique, and handling.^2-4^

The chronology in this case, onset within about 12 h, persistence over time, and worsening after re-exposure to another formulation, supports a delayed, likely T-cell–mediated hypersensitivity process rather than simple local irritation.^1^ This observation is clinically relevant because commercial botulinum toxin preparations differ in accessory proteins, excipients, and stabilizing agents, which may influence immunogenicity and tissue responses beyond the 150 kDa neurotoxin itself.^5-7^ Although the absence of immunological testing is an important limitation, the clinical pattern remains compatible with sensitization to one or more formulation components.^1^

Equally notable is the apparent response to OOT after corticosteroids and antibiotics had failed.^1^ Ozone has been investigated for its capacity to influence redox-sensitive signaling, local oxygen metabolism, inflammatory pathways, and tissue repair.^8,9^ Rather than simply increasing antioxidant enzymes and thereby reducing inflammation, a more precise interpretation is that controlled ozone exposure may generate a transient oxidative stimulus capable of activating adaptive cellular responses, including Nrf2-related pathways, with downstream effects on antioxidant balance and inflammatory signaling.^8-11^ This framing better reflects the complexity of physiological redox regulation and avoids an overly linear view of the relationship between oxidative stress and inflammation. In parallel, reported effects on hemorheology, oxygen delivery, and microcirculation may also have contributed to tissue recovery in this patient^8,9,12^

This case therefore contributes to 2 related discussions: the need to better understand the immunogenic potential of botulinum toxin formulations and the possibility that OOT may serve as an adjunctive strategy for modulating redox state, innate immune activity, and tissue healing in selected inflammatory complications.^1-12^
